# TANGO2 deficiency disorder in a 61-year-old male with episodic weakness, rhabdomyolysis, myotonia, and a novel missense variant

**DOI:** 10.1016/j.ymgmr.2025.101241

**Published:** 2025-07-04

**Authors:** Henry Skocy, Amber McFerren, Daniel Cairns, H. Mark Kenney, Eran Tallis, Amit S. Dhamoon, Ellie Garbade, Samuel J. Mackenzie

**Affiliations:** a School of Medicine, University of Rochester Medical Center, Rochester, NY, USA; b Department of Medicine, University of Rochester Medical Center, Rochester, NY, USA; c Department of Pediatrics, Division of Pediatric Genetics, University of Rochester Medical Center, Rochester, NY, USA; d Department of Neurology, University of Rochester Medical Center, Rochester, NY, USA

**Keywords:** TANGO2, Rhabdomyolysis, Neurometabolic, Mitochondrial disease, Muscle, Genotype-phenotype correlation

## Abstract

TANGO2 Deficiency Disorder (TDD) is an autosomal recessive condition, most commonly diagnosed in childhood. Clinical features may include episodic movement disorders, seizures, cognitive impairment, hypothyroidism, and metabolic crises marked by rhabdomyolysis and life-threatening cardiac symptoms. A small number of adults, thought to largely represent the milder end of the phenotypic spectrum, have received a diagnosis of TDD in their 30's or 40's, though no genotype-phenotype correlations have been established to date. In this case report, we present a 61-year-old man with mild intellectual disability and recurrent muscle weakness who was diagnosed with TDD during an inpatient hospitalization for diverticulitis, prostatitis, and muscle weakness, ultimately attributed to rhabdomyolysis. Genetic testing revealed a deletion of exons 3–9 in *TANGO2* along with a novel missense variant (c.187G > T; p.Gly63Cys) on the other allele. The patient was started on vitamin B-complex with additional pantothenic acid (500 mg daily) and subsequently noted improvement in his speech and energy levels. To our knowledge, this case describes the oldest known individual living with TDD by two decades. Additionally, the patient's relatively mild symptom profile and previously unreported missense variant in *TANGO2* may represent the first known example of genotype-phenotype correlation in TDD.

## Highlights

• TANGO2 Deficiency Disorder (TDD) is a rare autosomal recessive condition typically diagnosed in childhood
• This case report describes a 61-year-old man with TDD, the oldest known person living with the condition
• The p.Gly63Cys variant in *TANGO2* has not been previously described and may be associated with a less severe phenotype
• TDD should be part of the differential diagnosis for patients with episodic weakness, rhabdomyolysis, and myotonia

## 1 Introduction

Transport and Golgi Organization 2 (TANGO2) Deficiency Disorder (TDD) is a rare neurometabolic condition with an estimated global prevalence of approximately 1:1,000,000 [1] Symptoms arise following the inheritance of biallelic pathogenic variants in the *TANGO2* gene, which include multiexon deletions (most commonly exons 3–9) and point mutations [2,3]. TDD typically presents in infancy or early childhood and may include developmental delay and movement disorders as well as metabolic crises with rhabdomyolysis, hypoglycemia, and cardiac arrhythmias [[2], [4], [5], [6]] that tend to coincide with illness or fasting. B-vitamin supplementation, particularly pantothenic acid (vitamin B5), may have a therapeutic effect in TDD based on retrospective clinical data and work in disease models [[7], [8]]. As vitamin B5 is a precursor to coenzyme A (CoA), one theory holds that the CoA-mediated recovery of lipid homeostasis [[9], [10], [11], [12]] and the amelioration of mitochondrial dysfunction [[13], [14], [15]] underlie its benefit, but this has not been conclusively demonstrated.

Recent reports have highlighted the variable clinical expressivity in TDD, which may range from acute, severe decompensations early in life to more mild phenotypes without crises that often remain unrecognized into adulthood [3]. The oldest known patient with TDD based on published literature was diagnosed at age 40 after presenting with limb-girdle weakness and intellectual disability [16]. Herein, we present a case of a 61-year-old male with a longstanding history of intermittent weakness often preceded by illness, eventually prompting hospitalization in the setting of diverticulitis and poor oral intake. His history of recurrent weakness (typically managed at home) and evidence of atypically persistent creatine kinase (CK) elevations consistent with rhabdomyolysis prompted neuromuscular and genetic assessment, which led to a molecular diagnosis of TDD. This individual was found to have a novel missense variant in *TANGO2* and represents the oldest living patient with TDD by approximately 20 years. This case underscores the notion that TDD likely remains an underdiagnosed clinical entity, especially in the adult population, where symptoms may not consistently prompt an extensive diagnostic evaluation inclusive of genetic testing.

## 2 Case summary

### 2.1 Initial presentation

**Fig. 1:**
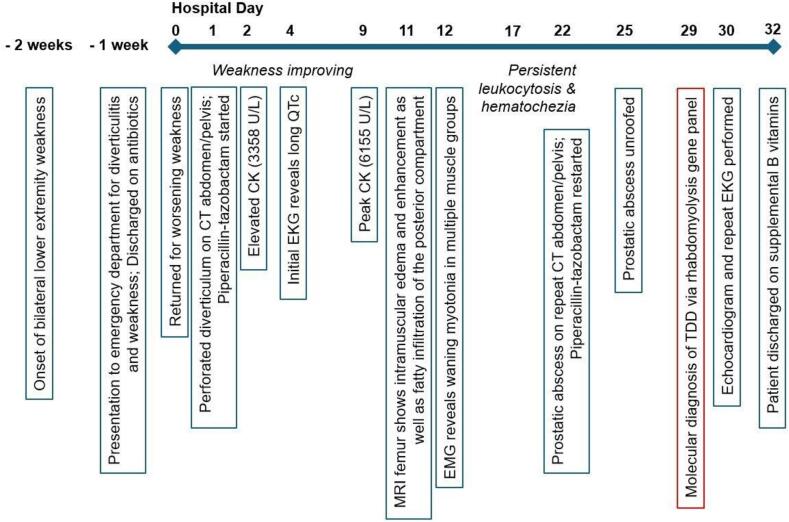
Timeline of patient's hospitalization. The patient developed lower extremity weakness prior to hospitalization in the setting of an upper respiratory infection. A diagnosis of TANGO2 Deficiency Disorder was established late in the patient's hospitalization course, prompting treatment with B-vitamin supplementation.

A 61-year-old Caucasian male with a history of learning disability and untreated psoriasis initially presented with subacute progressive bilateral lower extremity weakness and fatigue, accompanied by abdominal pain (Fig. 1). He reported a sudden onset of bilateral lower extremity weakness that started approximately 1–2 weeks prior to his hospitalization in the setting of an otherwise uncomplicated respiratory infection. The weakness progressed to the point that he required a walker to ambulate despite previously being able to do so without assistance. He denied pain or sensory changes in his extremities. He recalled experiencing similar episodes of acute bilateral lower extremity weakness throughout his life, sometimes requiring him to crawl around his house due to an inability to walk. He noted that at least two of these episodes were associated with upper respiratory infections, and both resolved without intervention. The patient's siblings, who helped provide his medical history, also noted new-onset slurring of speech superimposed on a more chronic history of halting speech and difficulty forming longer sentences. One week prior to admission, the patient had presented to the emergency department for periumbilical abdominal pain and non-bloody diarrhea. He was diagnosed with diverticulitis and discharged on a 10-day course of amoxicillin-clavulanate, but it was unclear whether he completed the full course.

The patient's medical history was significant for chronic psoriasis resistant to methotrexate and UV-light therapy. He did not take any medications for chronic conditions including his psoriasis, but he used aspirin as needed for diffuse joint pain and over-the-counter medications for seasonal allergies. He also reported a learning disability, and his siblings suspected he also had undiagnosed dyslexia. As a child, he was hospitalized once or twice around the age of two, from what his half-sister could recall. Records from this hospitalization could not be obtained, but she believed he experienced seizures and was given a working diagnosis of encephalitis at the time. He had recovered by the time he started elementary school and did not require further hospitalizations. The patient switched between special education and mainstream classes. He eventually graduated from high school and obtained a driver's license. Nonetheless, he struggled with certain tasks, such as paying bills, for which he required the assistance of his family. He worked as a dishwasher after graduation and more recently in environmental services but also experienced long periods of unemployment throughout his life. He lived with his mother until she moved into a nursing home four years prior to his presentation. At the time of his hospitalization, he had been living alone, with both of his siblings regularly checking in on him.

The patient also had muscular weakness and coordination issues in childhood, which made it difficult to play sports. His family described episodes throughout the patient's life where he would lie down and become unresponsive during illness. He reportedly underwent a muscle biopsy around age two in conjunction with his hospitalization, but the results of this test could not be obtained. The patient reported one notable instance 16 years ago in which he was unable to ambulate, ultimately requiring physical therapy to walk again. He noted that he did not regularly seek out medical care unless he had a serious issue that he did not self-resolve.

The patient's family history was significant for myocardial infarction and coronary artery disease in his late father, who died at age 50 from a stroke. There was no reported medical history in his mother, who was 96 and residing in a nursing home at the time of his presentation. One brother reportedly died at age 43 due to “heart problems” after undergoing multiple surgical removals of pancreatic growths. He also had a half-brother, age 67, living with renal cell carcinoma and another healthy half-sister, age 71. There was no known history of intellectual disability or delayed development in his family.

### 2.2 Early clinical course

On initial examination, the patient was noted to be morbidly obese (140.6 kg, 47.14 BMI) and non-distressed with normal vital signs. He had mildly slurred speech, dry mucus membranes, and signs of poor dentition. His left pupil was noted to be anisocoric and non-reactive to direct or consensual light. His abdomen was non-tender with normal bowel sounds. Erythematous, silver scaly plaques were present on the extensor surfaces of the upper and lower extremities bilaterally, as well as his back and lower abdomen. He had full strength in upper extremities and 4/5 strength with hip flexion, knee flexion, and knee extension.

**Fig. 2:**
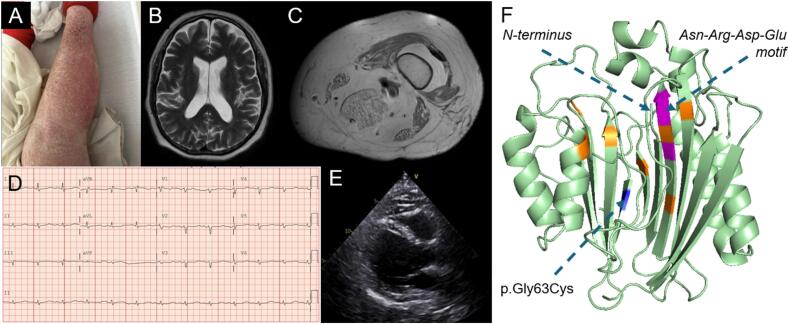
Extensive psoriasis of the bilateral upper and lower extremities (A). MRI head showed only mildly enlargement of ventricles and cortical fissures (B), but MRI of bilateral legs was notable for intramuscular edema and fatty atrophy of several lower extremity muscles (C). EKG demonstrated QT prolongation (QTc of 609 ms) (D) and echocardiogram showed concentric left ventricular hypertrophy (E). Genetic testing revealed a multi-exon deletion of exons 3–9 on one allele and a novel missense variant of unknown significance resulting in a glycine to cysteine substitution at amino acid 63 on the other allele (F, blue). This residue forms part of a beta sheet opposite a highly conserved Asn-Arg-Asp-Glu motif (magenta) within a putative hydrophobic binding pocket of the TANGO2 protein. Previously reported missense variants are depicted in orange. *In silico* model generated using AlphaFold-3 and PyMOL.

Initial labs demonstrated a leukocytosis (WBC 16,600 cells/μL) and elevated transaminases (ALT 224 U/L, AST 290 U/L). Computed tomography (CT) scan of the abdomen and pelvis demonstrated evidence of continued diverticulitis complicated by a focal perforation. Colorectal surgery was consulted, and medical management for diverticulitis was again recommended. The patient was initially treated with intravenous piperacillin-tazobactam, with a subsequent transition to a 14-day course of oral ciprofloxacin and metronidazole. He experienced intermittent hematochezia throughout his hospitalization but this resolved prior to discharge. His hospitalization was further complicated by a urinary tract infection and the development of a new prostatic abscess, which required restarting piperacillin-tazobactam and unroofing of the abscess. Post-operatively, the patient was transitioned to a 2-week course of trimethoprim-sulfamethoxazole. Additional findings during hospitalization included untreated chronic psoriasis (Fig. 2A), for which dermatology initiated topical triamcinolone therapy.

The patient's bilateral lower extremity weakness gradually improved over the early course of his hospitalization, although his strength remained below baseline and his siblings continued to express concerns of persistently slurred speech. His left pupil continued to be non-reactive and anisocoric, but the patient reported chronic issues with this eye. Nevertheless, with the slurred speech and nonreactive left pupil, magnetic resonance imaging (MRI) of the brain was performed. This study demonstrated mild enlargement of the sulci and fissures but did not show evidence of any acute process, such as infarction or hemorrhage (Fig. 2B).

Further laboratory studies noted a normal thyroid stimulating hormone (TSH) level (1.15 mIU/L) with elevated CK levels (3358 U/L on admission and peaking at 6155 U/L; Supplemental Table 1), elevated erythrocyte sedimentation rate (78 mm/h), and a normal gamma-glutamyl transferase level. The neurology, neuromuscular, medical genetics, and rheumatology teams were consulted due to concern for an underlying myopathic, metabolic or inflammatory syndrome given persistently elevated CK levels and history of episodic weakness provoked by illness. Autoantibody testing including ANA and RNP/Smith was recommended by rheumatology and was negative. An extensive myositis panel, including autoantibodies against Jo-1, PM/SCL-100, SUMO activating enzyme antibody, nuclear matrix protein-2, MDA5 (CADM-140) antibody, and IF-1 gamma antibody, was also negative. Additional laboratory studies recommended by medical genetics revealed a mildly elevated ammonia level (56 umol/L), abnormal acylcarnitine profile consistent with ketosis (Supplemental Table 2), abnormal urine organic acid profile (Supplemental Table 3), abnormal plasma amino acid profile (Supplemental Table 4), and urine uric acid of 24.7 mg/dL. Together, these results suggested a metabolic cause for the patient's weakness.Medical genetics initially recommended follow-up in the outpatient setting for genetic testing.

### 2.3 Imaging and diagnosis

To further evaluate the patient's weakness, the neuromuscular consult team recommended obtaining an MRI of the thighs, which revealed bilateral intramuscular edema and fatty atrophy of several lower extremity muscles (Fig. 2C). This atrophy included the semimembranosus and, to a lesser extent, the gracilis and sartorius muscles. A 3.4 cm lesion, consistent with an enchondroma, was also identified within the distal right femur. Nerve conduction studies and electromyography (EMG) were also obtained (Supplemental Table 5) and notable for electrical myotonia, most prominent in the proximal muscles. Trace percussion myotonia was observed in the finger extensors but not in the thenar muscles or trapezius. Additionally, the patient's EMG demonstrated early recruitment and subtle polyphasia in the right deltoid without overtly myopathic motor units. Without a history to suggest a drug-induced or toxic process, the findings were deemed suggestive of a metabolic myopathy. Multiple diagnoses were considered, including glycogen storage disease types II (Pompe disease) and IIIA (Cori disease), due to the episodic nature of the patient's weakness. Other etiologies including inclusion-body myositis and myotonic dystrophy were considered less likely, in part due to the recovery of leg strength in prior episodes.

An EKG demonstrated sinus rhythm with significant QT prolongation (QTc of 609 ms) and nonspecific intraventricular conduction delay (Fig. 2D). Since the patient's hospitalization was prolonged due to ongoing infections, medical genetics was reengaged to facilitate further testing while the patient remained admitted. A rhabdomyolysis and metabolic myopathy gene panel (Invitae) was obtained inpatient and ultimately identified a pathogenic multiexon deletion of exons 3–9 and a second variant of uncertain significance (c.187G > T; p.Gly63Cys) in the *TANGO2* gene. An echocardiogram revealed concentric left ventricular hypertrophy with an ejection fraction of 40–45 % and a mildly dilated right ventricle (Fig. 2E).

The patient was started on B-vitamin supplementation (B-complex containing 3 mg B1, 3 mg B2, 20 mg B3, 5 mg B5, 0.5 mg B6, 400μg B9, 1μg of B12, plus an additional 500 mg B5) per clinical consensus guidelines for TANGO2 Deficiency Disorder (TDD) [17]. By this time, his clinical status had improved considerably, prompting discharge from the hospital and outpatient follow-up with medical genetics and neuromuscular medicine. At discharge, the patient's CK level had normalized (21 U/L), and his EKG had markedly improved (QTc of 497 ms, borderline intraventricular conduction delay). Four months after discharge, he reported being back to his baseline health without recurrence of weakness. His siblings noted that his speech and energy levels seemed improved since starting B-vitamin supplementation. On examination, his speech retained a hypernasal quality but was not frankly dysarthric. He exhibited 4/5 weakness with hip flexion, extension, and adduction, but strength was 5/5 in all other muscle groups. Reflexes were hypoactive-absent throughout. On sensory examination, he had reduced pinprick and proprioception distally, possibly suggestive of a mild length-dependent polyneuropathy that was not detected on his prior electrodiagnostic testing. He required his arms to push up from a chair but noted that this was typical for him. Gait was slightly broad-based but without marked lordotic posturing or hip sway. The patient and his family were provided with an emergency protocol letter pertaining to metabolic crisis in TDD (https://tango2research.org/for-families/guidelines-er-protocol). A referral was placed for outpatient physical therapy and cardiology follow-up. A repeat EKG demonstrated normalization of his QTc interval (445 ms) with ongoing borderline intraventricular conduction delay.

## 3 Review

Since its discovery in 2016, our understanding of phenotypic heterogeneity in TANGO2 Deficiency Disorder (TDD) has evolved considerably. Common neurologic manifestations include cognitive impairment, developmental regression, ataxia, intermittent encephalopathic exacerbations, and various seizure types [7], but additional symptoms, including alternating hemiparesis [18], multifocal combined dystonia [19], congenital anomalies of the optic nerve [20], and paroxysmal sympathetic hyperactivity [21] have also been described. Hypothyroidism is a another poorly understood symptom of TDD. While common, it is not seen in all patients, and indeed, our patient had normal TSH levels during his admission. Cardiac complications may manifest in the context of metabolic crises [22], typically in the form of ventricular tachycardia and Torsades de Pointes following QT prolongation [[23], [24], [25], [26]]. Rhabdomyolysis is another hallmark feature of recurrent metabolic crises in TDD and ultimately led to the diagnosis in this case. To our knowledge, myotonia has not been described in patients with TDD, but few patients in the literature have undergone EMG. The fact that our patient had electrical myotonia and subtle percussion myotonia during his hospitalization suggests that TDD should be included in the differential diagnosis for this relatively rare electrodiagnostic finding. Additionally, the other symptoms that our patient experienced prior to his hospitalization (potential severe metabolic crisis in early childhood, intermittent unresponsiveness during childhood, mild intellectual disability, dysarthria) underscores the importance of diagnostic vigilance over the lifespan.

Recent work has demonstrated that timely intervention with B-vitamin supplementation may reduce symptom burden in TDD [[7], [27], [28]]. While the mechanism underlying this treatment response is not known, there are several working hypotheses related to the function of TANGO2 at the cellular level that may explain its benefit. Initially thought to play a role in the ER-Golgi function (Transport and Golgi Organization [29]), TANGO2 has more recently been shown to primarily localize to mitochondria [[9], [14], [30]], and its deficiency has been associated with dysregulation of lipid homeostasis [9], dysfunctional mitochondrial respiration, and increased production of reactive oxygen species [13]. Current guidelines specify that both vitamin B5 and B9 should be included as part of any supplementation regimen [17], though a recent case report in a child with TDD described therapeutic benefit from B5 treatment alone [27]. Since vitamin B5 (pantothenic acid) is an obligate precursor of coenzyme A (CoA), one possibility is that TANGO2 is required for CoA salvage, and that the cell becomes more reliant on *de novo* CoA synthesis in the absence of TANGO2. Alternatively, TANGO2 may have a more direct role in the mediation of redox homeostasis, leading to a secondary depletion of CoA in the TANGO2-deficient state. Clearly, more work is needed to resolve the mechanism of TANGO2 protein, clarify the optimal dosing regimen of B-vitamins, and determine to what extent dietary intake drives the variable expressivity of TDD. At present, we believe that a supplemental B-complex plus high-dose vitamin B5 at a minimum should be administered to patients with TDD, especially in light of the favorable risk-benefit profile.

Our patient's genetic testing revealed a known pathogenic deletion of exons 3–9 and a variant of unknown significance (c.187G > T; p.Gly63.Cys) in *TANGO2*. While parental testing was not performed, the codon associated with this highly conserved residue lies within exon 3 of the canonical *TANGO2* transcript, indicating that these variants occurred in *trans*. The resulting amino acid change lies within a beta sheet opposite a highly conserved Asn-Arg-Asp-Glu motif in a putative hydrophobic binding pocket of the TANGO2 protein, recently shown to bind acyl-CoA thioesters [7,30]. This missense variant has not been previously reported and is not listed in gnomAD. We speculate that it may be associated with a milder phenotype of TDD; however, functional studies and data from additional patients are required to validate this theory.

This case underscores the likelihood that TDD remains underdiagnosed at the population level, particularly for patients with milder phenotypes. We also believe the current literature related to TDD likely reflects this severity bias. For adults who may have missed the pediatric window of detection through genetic testing, development of screening tools that could guide genetic evaluation for older patients may increase diagnostic rates and inform our understanding of the true disease prevalence. Finally, while a diagnosis of TDD has historically been linked with negative perceptions related to quality of life for both patients and families [31], this case illustrates that vitality well into adulthood is possible for our patients. Indeed, as the standard-of-care for TDD continues to evolve, our hope is that positive long-term outcomes become the norm.

## 4 Conclusion

This report describes the oldest known patient with TDD by over two decades. Supporting observations from a previously published report of a 40-year-old woman with TDD [16], our patient's case suggests the existence of a milder disease phenotype and the notion that skeletal muscle involvement may be more prominent later in the disease course. Our patient was noted to have electrical myotonia on EMG, implicating TDD as a metabolic myopathy. Genetic testing revealed a previously undescribed missense variant in the *TANGO2* gene, and although additional investigation is required to confirm whether this variant is consistently associated with milder disease features, this could have critical implications for our understanding of structure-function relationships of TANGO2 at the protein level. Finally, as this case further extends the phenotypic spectrum of TDD, it also informs future epidemiological studies focused on the true incidence of TDD across the lifespan. Given the life-threatening nature of TDD and the favorable risk-benefit profile of B-vitamin supplementation, it is critical that we improve our diagnostic rates in children and adults alike.

## CRediT authorship contribution statement

**Henry Skocy:** Writing – review & editing, Writing – original draft, Visualization, Investigation, Data curation, Conceptualization. **Amber McFerren:** Writing – review & editing, Writing – original draft, Visualization, Investigation, Data curation, Conceptualization. **Daniel Cairns:** Writing – review & editing, Writing – original draft, Investigation, Data curation, Conceptualization. **H. Mark Kenney:** Writing – review & editing, Conceptualization. **Eran Tallis:** Writing – review & editing. **Amit S. Dhamoon:** Writing – review & editing, Supervision, Conceptualization. **Ellie Garbade:** Writing – review & editing, Supervision, Conceptualization. **Samuel J. Mackenzie:** Writing – review & editing, Writing – original draft, Visualization, Supervision, Investigation, Data curation, Conceptualization.

## Consent

Informed consent was obtained from the patient and his designated health care proxy prior to publication.

## Funding sources

None.

## Declaration of competing interest

SJM receives research funding from the TANGO2 Research Foundation, currently serves as chair of their Scientific Advisory Board, and is on their Board of Directors. The remaining authors declare no other conflicts of interest with regard to the content of this report.

## Data Availability

No data was used for the research described in the article.

## Appendix A Supplementary data

Supplementary material Image 1
