# Supplementary material for: TANGO2 deficiency disorder in a 61-year-old male with episodic weakness, rhabdomyolysis, myotonia, and a novel missense variant
Source: Mol Genet Metab Rep. 2025 Jul 4;44:101241. doi: 10.1016/j.ymgmr.2025.101241 (PMC12273206; doi:10.1016/j.ymgmr.2025.101241)
Supplement: Supplementary file 1 — Supplementary material [file mmc1.docx]

**5. Supplemental Materials**

**Supplemental Table 1:** Laboratory results. “HD” indicates the hospital day from day zero.

|  | **HD2** | **HD7** | **HD9** | **HD14** | **HD16** |
| --- | --- | --- | --- | --- | --- |
| **CK (U/L)** (39-308) | 3,358 | 5,101 | 6,155 | 1,770 | 907 |
| **ESR (mm/hr)** (0-20) | - | 78 | - | 87 | - |
| **ALT (U/L)** (0-50) | 220 | 342 | 462 | 336 | 240 |
| **AST (U/L)** (0-50) | 241 | 467 | 569 | 203 | 122 |

**Supplemental Table 2:** Acylcarnitine profile of patient on hospital day (HD) 4 and 5.

| **Acylcarnitine** (reference values in umol/L) | **HD4** (umol/L) | **HD5** (umol/L) |
| --- | --- | --- |
| **C2 (Acetyl)** (2.93 - 15.06) | 14.82 | **15.82** |
| **C3 (Propionyl)** (< = 0.82) | 0.74 | 0.70 |
| **C4 (Isobutyryl)** (< = 0.42) | 0.27 | 0.40 |
| **C5 (Isovaleryl)** (< = 0.24) | 0.26 | **0.32** |
| **C5-DC (Glutaryl)** (< = 0.23) | 0.10 | 0.15 |
| **C5-OH (3-OH-Isovaleryl)** (< = 0.07) | 0.04 | 0.05 |
| **C6 (Hexanoyl)** (< = 0.12) | 0.10 | **0.20** |
| **C8 (Octanoyl)** (< = 0.22) | 0.18 | **0.35** |
| **C8:1 (Octenoyl)** (< = 0.60) | 0.36 | 0.59 |
| **C10 (Decanoyl)** (< = 0.33) | 0.19 | **0.48** |
| **C10:1 (Decenoyl)** (< = 0.27) | 0.23 | **0.52** |
| **C12 (Dodecanyl)** (< = 0.13) | 0.06 | **0.14** |
| **C12:1 (Dodecenoyl)** (< = 0.13) | 0.10 | **0.22** |
| **C12 -OH (3-OH DOD)** (< = 0.02) | 0.01 | 0.02 |
| **C14 (Tetradecanoyl)** (< = 0.06) | 0.04 | 0.06 |
| **C14:1 (Tetradecanoyl)** (< = 0.15) | 0.14 | **0.24** |
| **C14:2 (Tetradecadienoyl)** (< = 0.08) | 0.08 | **0.12** |
| **C14-OH (3-OH-Tetradecaen)** (< = 0.01) | **0.02** | **0.02** |
| **C14:1-OH (Tetradecen)** (< = 0.04) | 0.02 | 0.03 |
| **C16 (Palmitoyl)** (< = 0.12) | **0.14** | **0.21** |
| **C16:1 (Palmitoleyl)** (< = 0.04) | **0.05** | **0.07** |
| **C16-OH, 3-OH-Palmitoyl** (< = 0.02) | **0.04** | **0.04** |
| **C16:1-OH,3-OH-Palmitoleyl** (< = 0.02) | <0.01 | 0.02 |
| **C18 (Stearoyl)** (< = 0.06) | 0.04 | 0.06 |
| **C18:1 (Oleoyl)** (< = 0.18) | 0.16 | **0.29** |
| **C18:2 (Linoleoyl)** (< = 0.10) | 0.08 | **0.12** |
| **C18-OH, 3-OH-Stearoyl** (< = 0.02) | 0.01 | 0.02 |
| **C18:1-OH (Oleoyl)** (< = 0.02) | 0.04 | **0.03** |
| **C18:2-OH (Linoleoyl)** (< = 0.02) | 0.02 | 0.02 |

**Supplemental Table 3:** Patient’s urine organic acid profile on hospital day 4.

| **Organic Acids**  (reference values in mmol/mol creatinine) | **Value**  (mmol/mol creatinine) |
| --- | --- |
| **Lactic Acid** (0 – 50) | 33 |
| **Pyruvic Acid** (0 – 15) | 19 |
| **Succinic Acid** (0 – 20) | 4 |
| **Fumaric Acid** (0 – 4) | 1 |
| **2-Ketoglutaric Acid** (0 – 75) | 9 |
| **Methylmalonic Acid** (0 – 5) | 1 |
| **3-OH-Butyric Acid** (0 – 4) | 276 |
| **Acetoacetic Acid** (0 – 4) | 219 |
| **2-Keto-3-Methylvaleric Acid** (0 – 10) | Not Detected |
| **2-Ketoisocaproic Acid** (0 – 4) | Not Detected |
| **2-Ketoisovaleric Acid** (0 – 4) | 1 |
| **Ethylmalonic Acid** (0 – 4) | 2 |
| **Adipic Acid** (0 – 35) | 32 |
| **Suberic Acid** (0 – 3) | 8 |
| **Sebacic Acid** (0 – 3) | Not Detected |
| **4-OH-Phenylacetic Acid** (0 – 25) | 49 |
| **4-OH-Phenyllactic Acid** (0 – 4) | 11 |
| **4-OH-Phenylpyruvic Acid** (0 – 2) | 2 |
| **Succinylacetone** (0) | Not Detected |
| **Creatinine (mg/dL)** | 234 (mg/dL) |

**Supplemental Table 4:** Patient’s plasma amino acid profile on hospital day 4.

| **Amino Acids** (reference values in umol/L) | **Value** (umol/L) |
| --- | --- |
| **Alanine** (160 – 530) | 206 |
| **Arginine** (35 – 125) | 48 |
| **Aspartic Acid** (< = 15) | <5 |
| **Citrulline** (10 – 45) | 34 |
| **Glutamine** (380 – 680) | 390 |
| **Glutamic Acid** (15 – 130) | 47 |
| **Glycine** (140 – 420) | 223 |
| **Histidine** (50 – 130) | 55 |
| **Hydroxyproline** (5 – 40) | 14 |
| **Isoleucine** (30 – 120) | 49 |
| **Leucine** (60 – 180) | 74 |
| **Lysine** (85 – 230) | 186 |
| **Methionine** (15 – 40) | 32 |
| **Ornithine** (25 – 110) | 72 |
| **Phenylalanine** (30 – 82) | 73 |
| **Proline** (90 – 350) | 172 |
| **Serine** (60 – 170) | 83 |
| **Taurine** (30 – 130) | 70 |
| **Threonine** (60 – 190) | 178 |
| **Tyrosine** (35 – 110) | 68 |
| **Valine** (120 – 320) | 145 |
| **Homocystine** (< = 2) | < 2 |
| **Allo-Isoleucine** (< = 5) | < 2 |
| **Cystine** (10 – 65) | 42 |
| **A-Aminobutyric Acid** (< = 40) | 30 |
| **A-Aminoadipic Acid** (< = 4) | < 2 |
| **Argininosuccinic Acid** (< = 2) | < 2 |
| **B-Aminoisobutyric** (< = 10) | < 5 |
| **B-Alanine** (< = 25) | < 25 |
| **Ethanolamine** (< = 15) | 8 |
| **G-Aminobutyric** (< = 5) | < 5 |
| **Sarcosine** (< = 5) | < 5 |
| **Tryptophan** (25 – 80) | 44 |
| **Anserine** (< = 5) | < 5 |
| **Asparagine** (20 – 80) | 50 |
| **Cystathionine** (< = 5) | < 5 |
| **Homocitrulline** (< = 5) | < 5 |
| **Hydroxylysine** (< = 5) | < 5 |

**Supplemental Table 5:** Nerve conduction studies and EMG performed on hospital day 12.

Motor Nerve Conduction Studies

| **Stimulation Site (Nerve)** | **Onset Latency** | **Amplitude** | **Conduction Velocity** | **Distance** | **Normal** |
| --- | --- | --- | --- | --- | --- |
| **Fibular head (fibular)** | 2.7 ms | 3.5 mV |  | 90 mm | yes |
| **Popiteal fossa (fibular)** | 4.3 ms | 2.7 mV | 56 m/s | 90 mm | yes |
| **Wrist (median)** | 3.6 ms | 8 mV |  | 70 mm | yes |
| **Elbow (median)** | 8 ms | 7.4 mV | 59 m/s | 260 mm | yes |
| **Wrist (ulnar)** | 2.8 ms | 10.8 mV |  | 70 mm | yes |
| **Below elbow (ulnar)** | 6.7 ms | 10.5 mV | 58 m/s | 225 mm | yes |
| **Above elbow (ulnar)** | 8.6 ms | 9.9 mV | 53 m/s | 100 mm | yes |

Sensory Nerve Conduction Studies

| **Stimulation Site (Nerve)** | **Onset Latency** | **Amplitude** | **Conduction Velocity** | **Distance** | **Normal** |
| --- | --- | --- | --- | --- | --- |
| **Wrist (median)** | 2.3 ms | 20 µV | 57 m/s | 130 mm | yes |
| **Wrist (ulnar)** | 1.93 ms | 16 µV | 57 m/s | 110 mm | yes |

Needle EMG

| **Muscle** | **L/R** | **Insertional Activity** | **Pos. Waves** | **Fasciculations** | **Duration** | **Amplitude** | **Phases** | **Activation** | **Recruitment** |
| --- | --- | --- | --- | --- | --- | --- | --- | --- | --- |
| **Trapezius** | R | Myotonia | 0 | 0 | Normal | Normal | Normal | Normal | Normal |
| **Deltoid** | R | Myotonia | 0 | 0 | Normal | Normal | 1 | Normal | Early |
| **Biceps Brachii** | R | Myotonia | 0 | 0 | Normal | Normal | Normal | Normal | Normal |
| **Extensor Digitorum Communis** | R | Myotonia | 0 | 0 | Normal | Normal | Normal | Normal | Normal |
| **First Dorsal Interosseous** | R | Myotonia | 0 | 0 | Normal | Normal | Normal | Normal | Normal |
| **Vastus Medius** | R | Myotonia | 0 | 0 | Normal | Normal | Normal | Normal | Normal |

*Myotonia noted to be waning and more prominent in proximal muscles
